# Desmoplastic Small Round Cell Tumor: A Review of Main Molecular Abnormalities and Emerging Therapy

**DOI:** 10.3390/cancers13030498

**Published:** 2021-01-28

**Authors:** Celso Abdon Mello, Fernando Augusto Batista Campos, Tiago Goss Santos, Maria Leticia Gobo Silva, Giovana Tardin Torrezan, Felipe D’Almeida Costa, Maria Nirvana Formiga, Ulisses Nicolau, Antonio Geraldo Nascimento, Cassia Silva, Maria Paula Curado, Suely Akiko Nakagawa, Ademar Lopes, Samuel Aguiar

**Affiliations:** 1 Department of Medical Oncology, A.C.Camargo Cancer Center, Sao Paulo 01509-010, Brazil; fernando.campos@accamargo.org.br (F.A.B.C.); nirvana.formiga@accamargo.org.br (M.N.F.); ulisses.nicolau@accamargo.org.br (U.N.); cassia.silva@accamargo.org.br (C.S.); 2 Laboratory of Tumor Biology and Biomarkers, International Center of Research CIPE, A.C.Camargo Cancer Center, Sao Paulo 01509-010, Brazil; tsantos@accamargo.org.br; 3 National Institute of Science and Technology in Oncogenomics and Therapeutic Innovation, Sao Paulo 05403-010, Brazil; giovana.torrezan@accamargo.org.br; 4 Department of Radiation Oncology, A.C.Camargo Cancer Center, Sao Paulo 01509-010, Brazil; leticia.silva@accamargo.org.br; 5 Genomics and Molecular Biology Group, International Center of Research CIPE, A.C.Camargo Cancer Center, Sao Paulo 01508-010, Brazil; 6 Department of Pathology, A.C.Camargo Cancer Center, Sao Paulo 01509-010, Brazil; felipe.costa@accamargo.org.br (F.D.C.); nascimento.antonio@accamargo.org.br (A.G.N.); 7 Department of Epidemiology, A.C.Camargo Cancer Center, Sao Paulo 01508-010, Brazil; mp.curado@accamargo.org.br; 8 Department of Surgery, A.C.Camargo Cancer Center, Sao Paulo 01509-010, Brazil; suely.nakagawa@accamargo.org.br (S.A.N.); ademar.lopes@accamargo.org.br (A.L.);

**Keywords:** desmoplastic small round cell tumor, treatment, prognosis, surgery, radiotherapy, chemotherapy, tyrosine kinase receptor, target therapy, rare disease

## Abstract

**Simple Summary:**

Desmoplastic small round cell tumor is a rare neoplasm with extremely aggressive behavior. Despite the multimodal treatment for newly diagnosed patients with chemotherapy, cytoreductive surgery and radiation, the cure rate is still low. For relapsed or progressive disease, there is limited data regarding second and third-line therapies. Novel agents have shown only modest activity. Recent molecular changes have been identified in this disease and this opens opportunities to be explored in future clinical trials.

**Abstract:**

Desmoplastic small round cell tumor (DSRCT) is an extremely rare, aggressive sarcoma affecting adolescents and young adults with male predominance. Generally, it originates from the serosal surface of the abdominal cavity. The hallmark characteristic of DSRCT is the EWSR1–WT1 gene fusion. This translocation up-regulates the expression of PDGFRα, VEGF and other proteins related to tumor and vascular cell proliferation. Current management of DSRCT includes a combination of chemotherapy, radiation and aggressive cytoreductive surgery plus intra-peritoneal hyperthermic chemotherapy (HIPEC). Despite advances in multimodal therapy, outcomes remain poor since the majority of patients present disease recurrence and die within three years. The dismal survival makes DSRCT an orphan disease with an urgent need for new drugs. The treatment of advanced and recurrent disease with tyrosine kinase inhibitors, such as pazopanib, sunitinib, and mTOR inhibitors was evaluated by small trials. Recent studies using comprehensive molecular profiling of DSRCT identified potential therapeutic targets. In this review, we aim to describe the current studies conducted to better understand DSRCT biology and to explore the new therapeutic strategies under investigation in preclinical models and in early phase clinical trials.

## 1. Introduction

**Figure 1:**
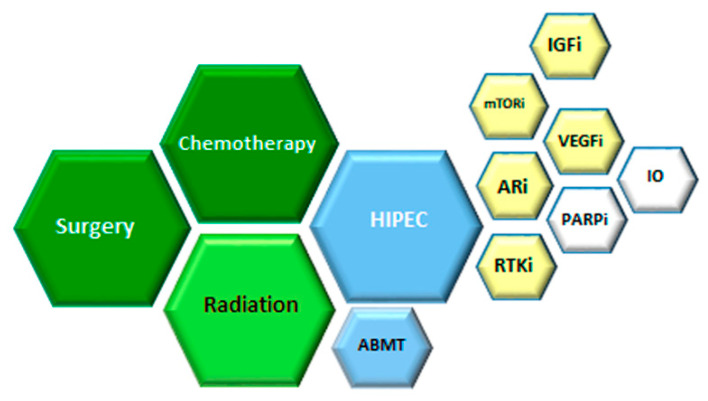
Therapeutic options for desmoplastic small round cell tumor (DSRCT). Based on multiple retrospectives and few prospective studies, the benefit was observed for therapies in green, low evidence of the benefit for therapies in blue. For relapsed or progressive disease, strategies in yellow had been used and in white are perspectives. HIPEC (hyperthermic intraperitoneal chemotherapy), RTKi (Receptor Tyrosine Kinase inhibition), ARi (Androgen Receptor inhibition), VEGFi (Vascular Endothelial Growth Factor inhibition), IO (immune check point inhibition), IGFi (Insulin Growth Factor inhibiotion), mTORi (mammalian Target of Rapamycin inhibition), ABMT (autologous bone marrow transplant).

Desmoplastic small round cell tumor (DSRCT) is an extremely rare, aggressive sarcoma. It affects mainly adolescents and young adults and originates in and primarily involves the serosal surfaces of the abdominal cavity. It was first described by Gerald and Rosai in 1989 as a newly characterized clinicopathologic entity [1]. Current management of the disease includes a combination of chemotherapy, radiation, and aggressive surgical resection [2,3] as summarized in Figure 1. Despite advances in multimodal therapy, the outcome remains poor since the majority of patients develop high rates of disease recurrence or die within three years [4,5]. Due to the dismal survival, DSRCT has an urgent unmet need for more effective and innovative therapeutic options.

### 1.1. Demographics of DSRCT

Desmoplastic small round cell tumor is a very rare subtype of sarcoma. For research purposes, DSRCT cases can be searched using the histology and behavior (malignant) classification code 8806/3. There is no uniform definition of rare sarcoma, however, the burden of rare cancer in our current days is great. The US Orphan Drug Act of 1983 defined rare diseases as those affecting less than 200,000 people in the United States [6]. In 2010, Greenlee et al. [7] described the US burden of rare cancers according to the National Cancer Institute definition as those cancers with fewer than 15 cases per 100,000 people per year. More recently, a consortium from the European Union, Surveillance of Rare Cancer in Europe (RARECARE) [8], described a new definition of rare cancer in Europe as those with fewer than 6 cases per 100,000 people per year.

In a study published in 2014 [9], a total of 192 cases of DSRCT were identified in the SEER database between 1973 to 2007. The age-adjusted incidence rate based on this analysis was 0.3 cases/million, with a peak incidence of 0.74 in individuals 20–24 years-old. There is a predominance of DSRCT in males. The age-adjusted incidence rates for males and females were 0.4 and 0.1 cases/million, respectively (*p* < 0.001) [9]. There is predominance in African–American individuals and it is more common in males. The age-adjusted incidence rates are higher among African-Americans as compared to Caucasians (0.5 × 0.2, *p* = 0.037, respectively). Out of 192 cases, the common primary sites of disease were the peritoneum or soft tissue of abdomen and pelvis (42%) and less common primary sites included the ovary/fallopian tube (6 cases), orbit (1 case), cerebellum (1 case), and cerebral ventricle (1 case) [9].

### 1.2. Molecular Profile of DSRCT

Cytogenetic and molecular characterization of DSRCT identified a unique chromosomal rearrangement, t(11; 22)(p13; q12), associated with this tumor [10,11]. The EWS-WT1 is the driver to tumorigenesis of DSRCT and it acts by up-regulating the expression of several genes [12]. The chimeric product of the EWS-WT1 fusion protein acts as a dominant transcriptional activator factor that regulates the expression of several growth factor genes, including *PDGFRA*, *IGF1R*, *EGFR*, *IL2*, *IL15* and also transcriptional factors such as *MYC*, *PAX2* and *WT1* [3,13].

**Figure 2:**
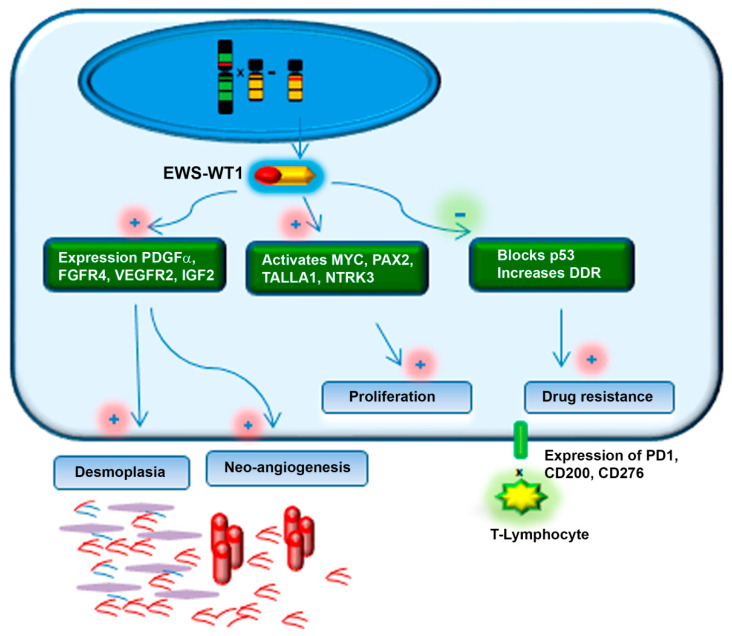
Schematic representation of EWS–WT1 fusion protein mechanism of action in desmoplastic small round cell tumor. Increase in tyrosine-kinase receptor expression, modulation of DNA replication proteins, activation of DNA-Damage Repair (DRR) machinery resulting in proliferation, desmoplasia, neo-angiogenesis and drug resistance.

The up-regulation of PDGFRα is a hallmark event in the development and DSRCT. The role of PDGFRα in the physiologic healing process is well described and is responsible for collagenous stromal production, inflammatory cell infiltration, especially macrophage chemotaxis and neo-angiogenesis [14], induces proliferation and is a chemo-attractant to fibroblasts and endothelial cells [14,15]. The development and growth of DSRCT is primarily dependent on this translocation product [12]. The EWS-WT1 transcription factor translocation produces a chimeric protein that induces the expression of PDGFRα that can explain the histological characteristics of DSRCT that is marked by profuse stromal proliferation and increased vascular density [16] (Figure 2).

Our group published a study with comprehensive molecular profiling of a patient with the diagnosis of DSRCT [17]. We identified genetic variants leading to protein alterations including 12 somatic and 14 germ-line events affecting genes predominantly involved in mesenchymal cell differentiation pathways. Regarding copy number alterations (CNA) few events were detected, mainly restricted to gains in chromosomes 5 and 18 and losses at 11p, 13q, and 22q. We developed a personalized test to follow up the patient and monitor disease recurrence by assessing the circulating tumor DNA (ctDNA) in the patient’s plasma. The genomic breakpoint of the EWS-WT1 gene fusion was tracked for the presence of minimal residual disease after surgery. This biomarker has been used in four post-treatment blood samples, three years after surgery, and no trace of EWS-WT1 gene fusion was detected, in accordance with imaging tests showing no evidence of disease and with the good general health status of the patient [17]. One interesting finding of our study is the fact that 7 out of 15 genes harboring somatic mutations (*CHL*, *MEGF10*, *MEIS2*, *MYH8*, *RIMS4*, *TBPL1* and *ZFPM2*) are regulated by the same transcription factor, *LEF1* (*p* < 0.001), which, in turn, is regulated by *WT1* [18]. We, therefore, postulate that DSRCT tumors presenting increased activity of *WT1* might up-regulate the expression of several genes mediated by *LEF1*. However, the accumulation of mutations in this set of genes regulated by *LEF1* and its relationship with the EWS-WT1 fusion protein remains to be addressed.

More recently, the molecular analysis [19] of 6 patients with DSRCT revealed a total of 137 somatic mutations which were related to specific biological processes: DNA damage-response (DDR) network and mesenchymal–epithelial reverse transition/epithelial–mesenchymal transition (MErT/EMT), reinforcing the relevance of these processes in tumor heterogeneity, aggressiveness and drug resistance [19].

There are many similarities between DSRCT and Ewing Sarcoma (ES) family tumors. Most of these tumors carry the EWS translocation. However, one important molecular aberration that distinguishes DSRCT from ES is the increased Androgen Receptor (*AR*) expression [20,21].

## 2. Clinical Presentation and Diagnosis

The disease predominantly originates from the peritoneum or retroperitoneum and can invade the omentum with multiple peritoneal implants involving the diaphragm, splenic hilum, mesentery of the small and large bowel, and the pelvic peritoneum [2,22,23]. Patients can be asymptomatic for long periods of time until symptoms of pain, ascites, constipation, weight loss, distension and jaundice [2,22]. Other sites of the primary tumor are described in the literature as the thoracic cavity, testicle, head and neck, intracranial, thigh, axilla/shoulder, intraosseous, uterine corpus, ovary, skull, middle ear, and others [24,25,26,27,28]. About one-half of the patients will present extra-peritoneal metastasis at the time of the diagnosis [9,22,29,30], although this percentage was lower in the report of Stiles et al. [23]. The liver and lung are the two most common sites for distant metastasis [2,3,23,29].

In most cases, patients with an abdominal disease are diagnosed in an advanced stage, with large masses and/or extensive seeding in the visceral and parietal peritoneum [30,31]. Symptoms, which are related to the tumor burden and location of the lesions, motivate investigation by image exams. The most common imaging finding is multiple, lobulated, low-attenuated, heterogeneous peritoneal, omental and serosal soft tissue masses usually discrete, round or ovoid, without an apparent primary organ of origin [32,33,34]. Almost all patients will present a dominant mass, mainly in the retrovesical or recto-uterine location, peritoneal or omental [33]. MRI can be helpful in delineating the extent of the disease, if surgical resection is considered [35] and can reveal lesions with heterogeneous contrast enhancement [36]. The role for position emission tomography (PET)-CT is not well established in DSRCT imaging, although it has been used each more as part of staging evaluation together with a chest CT scan [31,36]. There is no formal staging system for DSRCT [37]. One was proposed by Hayes-Jordan et al. [38] using the Peritoneal Cancer Index but it has not yet been validated.

Histologically, the tumor consists of solid sheets, large nests, small clumps, or cords of cohesive, small, round, ovoid, or spindled cells, with inconspicuous nucleoli, and scant cytoplasm, lying in a hypocellular, desmoplastic, collagenous stroma [39]. In immunohistochemistry, there is an expression of desmin, membrane antigen (EMA), cytokeratins (AE1/AE3 and CAM5.2) and neural markers (neuron-specific enolase and CD57), and smaller numbers expressing chromogranin, synaptophysin, CD56, neurofilament protein and S100 protein [40]. DSRCT can be immunoreactive for antibodies selectively directed to the carboxy terminus of the WT1 protein in more than 90% of cases [41]. It is important to note that DSRCT can show a polyphenotypic immune profile as well as a marked variation in morphologic appearances from tumor to tumor and within the same neoplasm [42].

## 3. Differential Diagnosis

The differential diagnosis of DSRCTs can be made with a spectrum of other round cell neoplasms, which includes ES, rhabdomyosarcoma, small cell carcinoma and mesothelioma [42]. Since the diagnosis of DSRCT is made by a combination of the histologic appearance and immunohistochemical staining results, it can be challenging in core biopsy specimens, once some of the distinctive features such as the prominent stromal pattern may not be easily appreciable, and atypical immunohistochemical features can be present due to the limited material [42]. DSRCT is distinguished from the other neoplasms by the presence of EWS-WT1 translocation, ideally performed by PCR or FISH or by immunohistochemistry staining if the former are not available. Nowadays, large fusion panels using RT-PCR are able to help in differentiating small round cell sarcomas from the ES family and new entities are been recognized such as tumors harboring *CIC-DUX4*, *BCOR-CCNB3* and *CIC-FOX04* fusions [43].

## 4. Treatment

### 4.1. Therapeutic Approach for Newly Diagnosed Patients

Multimodal therapy combining multi-agent intensive chemotherapy, aggressive debulking surgery and adjuvant radiotherapy is considered the standard of care for patients presenting without extra-abdominal metastases [29,44]. As most of the cases present as intra-peritoneal, aggressive tumors, the main guidelines recommend initiating treatment with systemic chemotherapy. Although DSRCT is not specifically addressed by ESMO [45] or NCCN guideline, this recommendation is stated by the Chicago Consensus on Peritoneal Malignancies [44] and by the MDAnderson Cancer Center nomogram [46].

The role of surgery in the management of DSRCT is well established in the literature. In a review of 12 patients treated at Mayo Clinic, Hassan et al. showed the median survival of patients treated with surgical resection was 34 months, whereas the median survival of those who underwent biopsy alone was 14 months [47]. In the report of Wong et al., the median survival for patients who had a resection for their abdominal or pelvic tumors was 47 months, compared to 16 months for those who did not [48]. Complete cytoreductive surgery is associated with improved survival and should be considered a cornerstone of treatment together with chemotherapy [46].

The most effective chemotherapeutic regimen with curative intent is still debated, but most are based on those used to treat other small round cell sarcomas, with a combination of an anthracycline, alkylating agent and vinca alkaloid. DSRCT is somehow sensitive to chemotherapy, although a transient response followed by disease progression is common [48]. Farhat et al. [49] reported four patients with the intra-abdominal disease who experienced disease stabilization lasting 4–9 months after chemotherapy including cyclophosphamide, etoposide, doxorubicin and cisplatin. Kushner et al. [50] reported 12 patients with a median survival of 19 months with the P6-protocol, which has seven courses of chemotherapy consisting of cyclophosphamide, doxorubicin, vincristine (HD-CAV), etoposide and ifosfamide. This was followed by surgery, radiotherapy, and myeloablative chemotherapy using thiotepa and carboplatin with stem cell rescue in some cases. Bertuzzi et al. [51] published a trial that included 7 patients with DSRCT treated with induction chemotherapy consisting of ifosfamide, epirubicin and vincristine, and those who responded were then treated with high-dose chemotherapy and autologous bone marrow transplantation in conjunction with local therapy (surgery and/or radiotherapy). The authors concluded that high-dose chemotherapy probably has no role in the treatment of DSRCT [51]. More recently, Scheer et al. [52] found that patients treated with the VAIA regimen (ifosfamide, vincristine, doxorubicin, actinomycin D) presented longer event-free survival (29.4 months) compared to other protocols, including the P6 protocol. The interval-compressed regimen of vincristine, irinotecan, temozolamide (VIT) was evaluated in 6 pediatric patients and showed a tolerable profile with an objective response rate of 50% to the first 2 cycles of VIT [53].

#### 4.1.1. Radiation Therapy

Given the propensity for morbid intra-peritoneal progression, consolidative whole abdominopelvic radiotherapy (WAP-RT) as part of multimodal treatment (chemotherapy, surgical debulking and WAP-RT) was first reported using the P6 protocol, in an attempt to improve local control [50]. Honoré et al. [29], reported in a series of 38 patients with a median follow-up of almost 5 years, that multimodal treatment combining systemic chemotherapy, complete macroscopic resection, and postoperative WAP-RT could prolong survival in patients without the extra-peritoneal disease (EPM)—median survival of 37.7 months (range 7.9–42.9 months). The factors predictive of 3-year overall survival were the absence of EPM, complete surgical resection, postoperative WAP-RT and postoperative chemotherapy [29].

Atallah et al. [54] studied the prognostic role of WAP-RT on oncologic outcomes as part of multimodal treatment in 103 patients with abdominal DSRCT treated at eight French centers from 1991 to 2014. Patients were retrospectively divided into three groups for evaluation: Group A treated with adjuvant RT after cytoreductive surgery, Group B without RT after cytoreductive surgery, and Group C treated with chemotherapy alone. Three-year OS was 61.2% (range 41–76%) in Group A, 37.6% (range 22–53.1%) in Group B, and 17.3% (range 6.3–32.8%) for Group C, respectively (*p* < 0.001). Peritoneal progression-free survival (PPFS) and progression-free survival (PFS) also differed significantly between the three groups (*p* < 0.001), but not distant progression. They concluded that RT seems to improve survival after cytoreductive surgery, with better PPFS, PFS and OS for the patients treated in a multimodal approach, with the limitations of a retrospective study, lack of statistical power (due to the small number of patients), and the need of randomized prospective studies to confirm these results [54].

In a more recent publication, Subbiah et al. [46] reported the MDAnderson Cancer Center experience with the treatment of 187 patients over two decades with the multimodal approach. The five-year OS rate was substantially improved from 5% to 25% with newer chemotherapy agents and better surgical and RT techniques. Chemotherapy response and complete cytoreductive surgery (CCS) were associated with improved survival. Their results also supported the use of WAP-RT when the time of diagnosis was used as a reference to estimate OS (univariate analysis, *p* = 0.01; HR, 0.44) [46]. However, because RT was given almost exclusively to patients who underwent CCS after chemotherapy, they removed these confounding factors and assessed the effects of WAP-RT using the date of the surgery as the start date in a time-variant analysis, and surprisingly, WAP-RT did not improve OS. This unexpected result conflicts with current clinical practice of a tri-modality therapy (chemo, surgery and radiation), and updated their treatment recommendation to consider WAP-RT in highly selected patients that are prospectively monitored in clinical trials [46]. Desai et al. [55] reported that acute toxicities of WAP-RT were primarily gastrointestinal and hematologic, and were improved in comparisons of IMRT against 2D-RT (gastrointestinal grade 2 or higher: 33% × 77%, *p* = 0.04; and grade 4 hematologic: 33% × 82%, *p* = 0.02), with no survival differences. Late toxicity (small bowel obstruction) did not statistically differ between RT modalities [55]. There are two main indications of radiation therapy in the control of DSRCT. One is in the palliative setting and the other is in an adjuvant scenario after complete cytoreductive surgery. The last one is the most recommended and adopted by most of the centers, despite clear evidence about the real prognostic impact is lacking, as seen in Table 1.

#### 4.1.2. HIPEC

Due to the rarity of this disease and analysis based on retrospective series, the role of radiotherapy in the management of DSRCT is still controversial. Appropriate patient selection is critical, as severe toxicities can occur. Despite aggressive multidisciplinary approaches, patients have a poor prognosis. Prospective randomized multicenter studies will be needed to evaluate the role of local treatments such as RT in the course of the disease.

Even after chemotherapeutic cytoreduction and surgical resection of gross, visible disease, the microscopic residual disease is often present [4]. Hence, hyperthermic intra-peritoneal chemotherapy (HIPEC) has been examined as an adjunctive intraoperative strategy. In a recent phase 2 trial, 14 DSRCT patients were treated with neoadjuvant chemotherapy, followed by cytoreductive surgery (CRS), which was complete (CR0) or near- complete (CR1 ≤ 2.5 cm of tumor remaining) in all patients, with closed technique HIPEC using 100 mg/m^2^ of cisplatin for 90 min at 41 degrees Celsius, then followed by WAP-RT [58]. The 3-year overall survival from time of diagnosis for DSRCT patients was 79%, and the estimated median recurrence-free survival (RFS) was 14.0 months. In 100% of patients without hepatic or portal metastasis, there was no peritoneal disease recurrence after CRS-HIPEC. They concluded that CRS, HIPEC and WART are effective local control therapy in DSRCT patients. Earlier, it was demonstrated that patients who had CR0 or CR1 and HIPEC had significantly longer median survival compared with patients who had HIPEC and gross residual disease greater than 2.5 cm after surgical cytoreduction (63.4 vs. 26.7 months) [59]. In an important retrospective study conducted at MDAnderson Cancer Center, most of the patients (72%) received combined treatment with chemotherapy, surgery plus HIPEC and radiation therapy. This study was underpowered to conclude about the impact of HIPEC on the OS. However, there was not found improvement in the three- and five-year OS (*p* = 0.12) of patients treated with HIPEC. Patients with DSRCT and disease outside the abdomen at the time of surgery do not benefit from HIPEC [59]. Until now, there is no randomized trials designed to evaluate the relative contribution to improve outcome after complete surgical excision of intra-abdominal implants.

A retrospective study with 187 DSRCT patients confirmed that chemotherapy and CCS remain the cornerstone of treatment, and suggest that prospective randomized studies will be required to prove the unequivocal benefit of HIPEC or WAP RT in the management of DSRCT [46].

### 4.2. Prognosis

Despite multimodal treatment, DSRCT has a poor prognosis, and approximately 60–70% of patients die due to disease progression usually within 3 years after diagnosis [5,23,58,60]. The median overall survival varies between 28–60 months, with a median disease-free survival between 10–15.5 months [23,29,57,58,60]. Table 2 shows a comparison of multimodal treatment in DSRCT.

One study proposed that the absence of extra-peritoneal metastasis, complete surgical resection and postoperative WAP-RT are factors predictive of 3-year overall survival [29]. The multimodality treatment combining chemotherapy, cytoreductive surgery, HIPEC and WAP-RT can warrant local control of the disease, but patients will still present distant metastasis during follow-up, meaning that more effective chemotherapy is necessary to improve long-term outcomes [58].

### 4.3. Current and Emerging Therapy for Relapsed or Progressive Disease

DSRCT is characterized by poor response to conventional chemotherapy and early relapse after radical surgery. Second-line treatment is ineffective in most cases. In our cohort, out of 19 patients treated with first-line chemotherapy, 13 received the second-line and the progression-free survival was only 3.9 months [57]. This short survival time highlights the aggressiveness of this disease and the challenge in developing new therapeutic strategies to treat these young patients. Despite the development of new regimens for ES and other soft tissue and bone sarcoma in recent years, DSRCT is underrepresented or were not included in the trials that lead to the drug approval [62]. As a result, the evidence to use second-line therapy is very limited. It is paramount to develop active cooperative groups to quickly collect data and propose new strategies for the treatment of DSRCT. Moreover, patients outside Europe and North America are almost never offered the opportunity to participate in clinical trials for rare diseases. The SELNET (https://selnet-h2020.org/), that is a Horizon 2020/EU project, aims to create a network between European and Latin American countries to improve diagnosis and treatment of sarcomas and eventually to develop clinical trials in these centers across the Atlantic Ocean. Table 3 displays the outcome of main studies with medical treatment and explores the response rate according to the line of treatment.

#### 4.3.1. The Importance of Pre-Clinical Models to Drug Development for Rare Sarcoma

The use of preclinical models is an important step in the development of new therapies for tumors in general and is crucial for rare tumors such as DSRCT. Due to the rarity of DSRCT, conducting clinical trials with new drugs is extremely difficult for many reasons [74]. First, the fact that genetic and functional comprehensive analyses of these tumors are limited. Second, the aggressive behavior and chemotherapy resistance makes the inclusion in target therapy clinical trials difficult. Accrual of patients in clinical trials is difficult due to the limited number of individuals affected yearly. As a result, the use of pre-clinical models is an important step in the development of novel drugs since a higher number of mechanisms can be modulated and faster therapeutic targets can be explored. Modeling these tumors with experimental models allows the investigation of the molecular mechanisms that underlie tumor origin and progression. The fidelity of the model is also related to the predictive capacity to anticipate eventual effects of drugs which will help to determine the efficiency and efficacy of anti-tumoral drugs. In the case of rare tumors, the possibility of scaling-up is crucial to translate technology from the bench to the bedside.

In 2002, Nishio and collaborators [75] reported for the first time the development of the DSRCT cell line, named JN-DSRCT-1, derived from pleural effusion from a 7-year-old patient with lung metastasis. JN-DSRCT-1 cells are small, round or spindle-shaped with oval nuclei and were maintained continuously in vitro for more than 190 passages for more than 40 months. The cell has a tumorigenic capacity and the histology of heterotransplanted tumors in SCID mice maintains the characteristics of the original DSRCT, including the expression of immunohistochemical markers (vimentin, desmin, CD57, among others), a t(11; 22) translocation (p13; q12) and presence of EWS-WT1 fusion [75]. JN-DSRCT-1 cells are being used in several studies that help to unveil DSRCT biology, especially the role of EWS-WT1 fusion protein and also to identify targets for therapeutic interventions [12,76].

As mentioned before, *EWS*-*WT1* translocation is the major driver in DSRCT and plays many roles in tumor biology that have the potential to be used as therapeutic targets. As already described in other studies, *EWS*-*WT1* gene underwent RNA splicing and one variant lacks three amino-acids and was named *EWS*-*WT1*(-KTS) due to the absence of Lys-Thr-Ser residues [77]. This isoform activates a gene encoding a tetraspanin-family protein, T-cell acute lymphoblastic leukemia-associated antigen 1 (*TALLA-1*) [78]. *TALLA-1* is part of multi-protein family involved in several processes, such as cell adhesion, migration and metastasis, and this gene could be a candidate for diagnostic marker and a putative target for therapy [78]. Another target for *EWS*-*WT1* fusion gene is *ENT4* (equilibrative nucleoside transporter 4) which encodes a pH-dependent adenosine transporter [79]. Neural gene induction is also triggered by *EWS*-*WT1* in JN-DSRCT-1 cells and neural reprogramming factor *ASCL1* is an important player in mediating multiple WT1-responsive elements, suggesting that neural differentiation pathway could be tested as therapeutic agents for DSRCT [80]. Recent work described the dependence of *EWS*-*WT1* in DSRCT survival [12]. Silencing EWS-WT1 causes proliferation loss, growth arrest and gene expression analysis indicates repression of estrogen signaling and highlights therapeutic genetic vulnerabilities, such as *FGFR4*, *JAK3*, *mTOR*, *PDGF*, *ERG*, and TGFB1 genes [12]. Another study that evaluated potential therapeutic targets performed RNA sequencing of 12 tumor samples from pediatric patients with DSRCT found high expression of *IGF2*, *FGFR4*, *CD200* and *CD276*, the latter two molecules are candidates for immune checkpoint inhibitor therapy [81].

In terms of therapy, the first use of JN-DSRCT-1 cells was to test the effect of rapamycin-induced apoptotic death [82]. The mechanism involves the up-regulation of Bax concomitant Bcl-xL down-regulation. Rapamycin also down-regulates *EWS*-*WT1* and 26S p44.5 proteasome subunit, suggesting that rapamycin induces apoptosis by preventing the degradation of the Bax protein by the proteasome, and that this process is independent of mTOR inhibition. Furthermore, these results strongly support the introduction of the use of rapamycin as a cytotoxic agent for the treatment of DSRCT. JN-DSRCT-1 cells were tested to verify the effectiveness of the TRAIL receptor agonist (apoptosis inducer), called ONC201 [83]. In this study, it was found that the induction of TRAIL decreases proliferation and induces apoptosis in vitro and decreases tumor growth in vivo. The potential of anti-angiogenic agents in decreasing tumor growth of the JN-DSRCT-1 cell was also investigated [74]. Animals with JN-DSRCT-1 cell xenografts were treated with bevacizumab and showed a prolongation of the time to progression and the long-term regressions were marked after treatment with the combination of irinotecan and bevacizumab compared to irinotecan alone [74]. Interestingly, there is recent evidence that indicates that the use of other anti-angiogenic agents may be effective in the treatment of DSRCT, as in the case report of a patient with advanced DSRCT, in the second-line of treatment, refractory to cisplatin, was treated with apatinib (VEGFR-2 inhibitor drug) and had a positive response, with a significant reduction in tumor mass [84]. JN-DSRCT-1 cells are sensitive to alkylating agent trabectedin and the mechanism of action involves the expression of genes involved with proliferation and apoptosis [85]. An alternative mechanism of action of trabectedin is the impairment of transactivation of *FUS*-*CHOP* fusion protein in liposarcoma [86]. This activity is also observed in *EWS*-*WT1* fusion protein, trabectedin reduces its binding on its target gene promoters and, thus, affects *EWS-WT1*—dependent gene expression in JN-DSRCT-1 cells [85]. Recently, the combination of PARP inhibitor olaparib with the alkylating agent temozolomide was tested in JN-DSRCT-1 cells in vitro and in vivo and the results indicates that the combination has synergistic effects upon cell viability, inducing cell cycle arrest which progress to apoptosis induction, causing tumor reduction [76].

Additionally to JN-DSRCT-1 cells, Markides and collaborators established more DSRCT cell lines, including BER lineage that also presents *EWS*-*WT1* fusion protein and have similar behavior of JN-DSRCT-1 [12,87]. Together, this evidence shows the importance of obtaining tumor models to accelerate preclinical research, bring possibilities for investigating new therapeutic approaches for this rare but lethal malignancy.

#### 4.3.2. Targeting Angiogenesis and Other TKR

As a hypervascular tumor, DSRCT is characterized by an overexpression of proteins that promote and maintain the angiogenic process necessary for continued tumor growth and proliferation. *EWS*-*WT1* is able to induce *PDGFA* expression [21] and activation of the *IGF1R* gene [14] (Figure 2). Other tyrosine kinase receptors (TKR) expression have been found to be disrupted in DSRCT and are related to proliferation and angiogensis. VEGFR-2 and VEGFA expression was found to be markedly increased in the DSRCT tumor sample and in the human DSRCT cell line, JN-DSRCT [88].

The use of tyrosine kinase inhibitors (TKI) for VEGF, VEGFR, PDGFRα and other proteins involved in tumoral vascular proliferation has been explored in the clinical scenario [84,89,90]. Pazopanib, apatinib and sunitinib inhibit angiogenesis by abrogating the VEGF-induced phosphorylation of VEGF receptors as well as other TKRs including PDGFR, FGFR, and c-KIT, affecting downstream activation of the PI3K/AKT, PKC, and other pathways that mediate cell proliferation, migration, and survival [91].

In the PALETTE study, 369 patients were randomized to receive pazopanib 800 mg/day versus placebo [62]. Median PFS was 4.6 months (95%CI: 3.7–4.8) for pazopanib compared with 1.6 months. A combined analysis of patients with the diagnosis of DSRCT treated in the EORTC phase II study 62,043 (3 patients), EORTC phase III 62,072 (3 patients) and in a UK Pazopanib expanded access program (3 patients) was performed [89]. Data from nine patients included in this analysis revealed a median age of 30 years and all patients were males with widespread metastatic DSRCT. Four patients had one previous chemotherapy line (44%), four had 2 previous chemotherapy lines (44%) and one patient 3 (12%). The response rate was partial response (PR) in 2/9 (22%) patients, stable disease (SD) in 5/9 patients (56%) and progressive disease (PD) in 2/9 (22%) with a clinical benefit rate (PR + SD > 12 weeks) of 78%. Median PFS and OS were 9.2 (95% CI: 0–23.2) and 15.4 (95% CI: 1.5–29.3) months respectively [89]. In a relatively large, retrospective study with 29 patients treated with pazopanib, clinical benefit was observed in 62% (18/29) of patients with DSRCT (CR in 1 patient, PR in 1 patient, SD in 16 patients) and the median progression-free survival was 5.4 months [71].

Sunitinib was one of the first generation of TKIs with great inhibition of VEGF receptor 2 among other TKRs. In vascular sarcomas, sunitinib showed early and promising activity in alveolar sarcoma, a chemo-resistant subtype of sarcoma [92]. In a retrospective analysis of patients with DSRCT, sunitinib showed clinical benefit in 8 patients evaluated [69]. Partial response was observed in two patients and SD in 3 patients treated in the second-and-beyond-line of therapy. Sorafenib was used in 2 patients [90], both in the fifth-line of therapy. The best response to sorafenib was stable disease and the median progression-free survival of 3.5 and 4 months. On the other hand, as first-line treatment, apatinib was used in only one patient [84]. Apatinib is another VEGFR-2 inhibitor with demonstrated activity in gastric and other tumors [93]. Clinical benefit and tumor shrinkage were reported in one patient treated with apatinib in the first-line. The patient had not received chemotherapy previously [84]. Another report showed partial response with apatinib in combination with chemotherapy as second-line treatment [94]. Ramucirumab, a VEGFR inhibitor is been tested in combination with cyclophosfomide and vinblastine in patients with relapsed and refractory DSRCT (ClinicalTrials.gov Identifier: NCT04145349).

Bevacizumab, a VEGF-A inhibitor, was combined with irinotecan and temozolamide (ITB regimen) in the first-line treatment of DSRCT. In this single-arm pilot study, 14 out of 15 patients completed the planned treatment that comprised two cycles of ITB followed by the conventional trial P6 with VAC and IE. The response rate to ITB was 27% and no major unexpected adverse event was observed [64].

Most of the trials with novel agents are designed to treat a myriad of histologies, including DSRCT. It is difficult to identify trials accruing only patients with this disease. We provide a summary with selected ongoing trials accruing patients with, but not limited to, DSRCT, displayed in Table 4.

#### 4.3.3. Targeting Androgen Receptor Pathway

The increased prevalence of DSRCT in young males motivated the investigation of the testosterone synthesis pathway in tumorigenesis of this disease. In 2007, Fine et al. [95] first demonstrated AR expression in DSRCT. In a cohort of 27 heavily pretreated patients, 37% stained positive for AR. The functionality of the pathway was demonstrated by in vitro assay that showed growth of tumor cells when stimulated by di-hydro-testosterone, and inhibition of growth by flutamide [95]. Another study performing single-sample gene set enrichment analysis found that the majority of DSRCTs were enriched for the AR signature when compared to other sarcomas, such as ES and alveolar rhabdomyosarcoma [3,20].

In the previous reported Fine et al. [95] study, six patients with AR-positive DSRCT received combined androgen blockade (CAB) with bicalutamide and leuprorelin. Three patients had clinical tumor benefits for a period lasting 3 to 4 months. All of them had normal testosterone levels at the initiation of CAB therapy, while the other three non-responders had castrated levels. In another report, a patient with strong AR expression received anti-androgen therapy with bicalutamide, presenting progressive disease 2 months later [73]. Negri et al. [21], using whole genome gene expression profiling and a cancer stem cell gene array, showed that AR-positive DSRCT cells harbor characteristics of stemness, which could explain the limited effectiveness of targeting this pathway.

#### 4.3.4. Targeting PI3K/AKT/mTOR Pathway

Activation of the phosphatidylinositol-3-kinase (PI3K)-protein kinase B (Akt)-mammalian target of rapamycin (mTOR) pathway is proposed to be implicated in the development of a variety of sarcomas [96,97,98]. There is emerging data indicating the possible involvement of the mTOR pathway in DSRCT. A single-center small study attempted to evaluate the morphoproteomic profiling of the mTOR pathway in DSRCT, ES and Wilms’ tumor, and showed that the PI3K/Akt/mTOR pathway is constitutively activated in DSRCT [99]. Another study described a patient with DSRCT harboring a secondary somatic mutation in the *PIK3CA* gene [100].

In vitro study demonstrated that rapamycin, an mTOR inhibitor, induced the apoptotic death of DSRCT line cells [82]. There are few data on the clinical efficacy of inhibition of PI3K/AKT/mTOR pathway. In one case report, a 21-year-old man with DSRCT achieved stable disease with temsirolimus for 40 weeks [73]. Tarek et al. [72] reported their experience with five patients with relapsed DSRCT treated with vinorelbine, cyclophosphamide and temsirolimus, all of them presenting partial response, with median time to progression of 8.5 months (range 7–16 months). A phase I trial evaluated the combination of cixutumumab (an IGFR antibody) with temsirolimus, which resulted in stable disease lasting longer than 5 months in two of the three patients with DSRCT of the study [101]. In a retrospective series of patients with high-grade STS treated with pazopanib plus sirolimus following progression on pazopanib, one patient with DSRCT had stable disease for 11 months with the combination treatment [102]. Recently, a trial was designed to estimate the response rate to two initial courses of temsirolimus, temozolomide and irinotecan (window therapy) in previously untreated patients with high-risk ES family of tumors, including DSRCT (ClinicalTrials.gov Identifier: NCT01946529). The interim analysis determined the window therapy did not meet the anticipated response, and trial accrual was stopped.

#### 4.3.5. Targeting DNA Damage Repair (DDR) Proteins

Studies using next-generation sequencing characterized a subgroup of DSRCT with secondary genomic alterations in genes associated with DNA damage repair (DDR), including *ATM*, *RAD50*, *BARD1*, *BRCA1/2*, *PALB2* and *CHEK2* [19,103]. It is still unknown if those genomic alterations act as driver mutations in DSRCT tumorigenesis [103].

Since poly(ADP-ribose) polymerases (PARPs) perform an important role in DDR, specifically in the base excision repair of single-strand DNA breaks, PARP inhibitors recently emerged as a new treatment for cancer-based on synthetic lethality concept, particularly in *BRCA*-mutant cancers defective in homologous repair [104,105]. DSRCT has high-level of PARP1, the most abundant enzyme of the PARP family, and a combination of olaparib and temozolamide has demonstrated enhanced antitumor effects in vitro and in vivo [76,106,107].

It was described that *EWS*-*FLI1* in ES and *EWS*-*WT1* in DSRCT might share common mechanisms of gene expression de-regulation [100]. *EWS-WT1* up-regulates the expression of *ERG*, an ETS family member of FLI1. It is possible that *ERG* may drive the expression of these targets, making this tumor an ETS-like tumor [15]. Another characteristic observed in vitro is that the tumor modulates the DNA damage response, both suppressing p53 signaling and driving the expression of gene sets associated with the DNA damage response, suggesting a direct link to the resistance to chemotherapy in DSRCT [12]. Deregulation of DNA damage response is another important feature of the other FET family fusions such as *EWS-FLI1* in ES. This characteristic makes the use of PARP inhibitor an attractive therapeutic strategy in both ES and DSRCT. The combination of trabectedin and PARP inhibitor is under investigation in clinical trials. Trabectedin is an intercalating DNA agent that promotes DNA damage and deregulate several repair pathways, inhibits transcriptor factors such as *FUS-CHOP* factor a exert cytotoxic activity in certain subtypes of cells such as the tumor-associated macrophages (TAM), myxoid liposarcoma and ES [108,109]. The combination of trabectedin and olaparib has shown robust inhibition of tumor proliferation in sarcoma mouse models [110]. The heavy damage in the single strand or double-strand DNA was not repaired by olaparib in the experimental model [110]. A phase Ib trial (TOMAS) conducted by the Italian Sarcoma Group was designed to explore the synergistic effect of trabectedine and olaparib in patients with advanced sarcoma and showed promising results [111]. Out of 50 accrued, 11 had a diagnosis of bone tumor and of these only 4 with ES and no activity was observed in this group of patients, despite the biological rationale for this combination in ES. The efficacy of this combination is under evaluation by the phase II trial TOMAS2 (ClinicalTrials.gov Identifier: NCT03838744).

In an ongoing trial, prexarsetinib, an inhibitor of checkpoint kinase 1 (chk1) in combination with irinotecan and temozolamide is currently under evaluation in an early phase trial (ClinicalTrials.gov Identifier: NCT0409522).

#### 4.3.6. Targeting c-MET and Insulin Growth Factor Pathway

c-Met (mesenchymal-epithelial transition factor) has been found to be overexpressed in a variety of solid tumors, including sarcomas [112,113,114,115], but the involvement of this receptor in DSRCT development is still scarce [100]. In the largest DSRCT comprehensive genomic profile study, no secondary mutation on *c-MET* was found [103]. There is a case report of a patient with intra-abdominal DSRCT who received anlotinib, a multi-kinase inhibitor that targets c-Met, for the progressive disease after surgery and first-line chemotherapy showing stable disease for 4 months [116].

The oncogenic fusion product EWSR1-WT1 in DSRCT was reported to activate the IGF-1R gene promoter, providing the basis to test the activity of anti-IGF-1R antibodies in the metastatic setting [117]. IGF2 has been up-regulated by the fusion product and is a potential target [81]. In a phase II study, ganitumab administered to 16 metastatic DSRCT patients determined one PR (6%) and 10 (63%) SD, with a median PFS of 15 months [66].

#### 4.3.7. Cancer Vaccines

Although initial studies on cancer vaccines had shown disappointing results with low response rates, a better understanding of the interaction between tumor, microenvironment, and immune system in the last decades have opened new perspectives for this therapy, including in the sarcoma field [118,119]. Cancer vaccines seek to induce tumor immune responses through antigen presentation and stimulation of new T cell responses [118]. Few studies have explored vaccines in DSRCT treatment. A phase I study using a vaccine of tumor lysate-pulsed autologous dendritic cells in the treatment of pediatric patients with solid tumors demonstrated feasibility for generating specific T-cell responses and regression or stabilization of metastatic disease in some patients, but failed to prevent progressive disease in the patient with DSRCT included in the trial [120]. A more recent trial evaluated the efficacy of an adjuvant dendritic cell vaccine administered three to eight weeks after completion of standard treatment in pediatric patients with high-risk sarcomas [121]. The survival advantage was demonstrated for patients with ES and rhabdomyosarcoma, but no clinical benefit was seen in the two patients with DSRCT in the study [121].

#### 4.3.8. Perspectives with Novel Targets (Immune Checkpoint and NTRK Inhibitors)

In general, sarcomas are not considered good candidates for immune therapy the way this therapy has currently been applied for other tumors [122]. Around one-third of sarcomas are characterized by single gene translocation that acts as a driver mutation. Moreover, sarcomas are amongst the neoplasms with the lowest tumor mutational burden, a recognized predictor of response to immune check point inhibitors (IO) [123].

Initial data using the combination of anti-PD1 +/− anti-CTLA-4 have shown promising results, particularly in alveolar soft part sarcoma (ASPS), undifferentiated pleomorphic sarcoma (UPS) and dedifferentiated lipossarcoma [124,125]. The ALLIANCE trial demonstrated that nivolumab monotherapy is not effective with only 5% of objective response rate against 16% for the combination of nivolumab and ipilimumab [125]. In the SARC28 trial, pembrolizumab induced objective response rate in 5 UPS patients [124] and a post hoc analysis of this trial demonstrated that patients with a specific sarcoma immunological classification (SIC) based on gene expression analysis derived the greatest benefit from pembrolizumab. This immune “high” or “hot” signature was associated with the structure of T lymphocyte, follicular dendritic cells and enriched B cells [126]. The combination of immune check point inhibitor and TKIs was evaluated and showed a safe profile and promising activity in a phase II trial with predominant ASPS patients [127]. Out of 11 patients with ASPS, six achieved a partial response (54.5%, 95% CI 24.6–81.9), and two (18%) of 11 achieving stable disease with a median PFS of 12 months [127].

It is difficult to predict that IO will be active in DSRCT based on preliminary data about immune regulation and biomarkers expression in this rare disease. Gene expression analysis revealed that DSRCT is characterized by a signature of immunological ignorance [21]. However, a study with samples of paraffin embed tumor sample showed elevated PD1 expression by tumor cells [128]. The expression of PD1, PDL-1, and CD8 was analyzed in a cohort of 11 patients with DSRCT and it was observed a high rate of PD1 (81%), CD8 (64%) and low PDL-1 (18%) expression [128]. Additionally, in vitro assay using the JN-DSRCT-1 cells culture to test the activity of nivolumab showed no effect in decreasing tumor cell proliferation. It is in line with the most recent data that demonstrated the single-agent nivolumab was ineffective for most sarcoma subtype [125]. On the other hand, the combination of an anti-PD1 and anti-CTLA4 (ipilimumab) resulted in a better outcome [129]. However, preclinical data using gene expression analysis showed that DSRCT overexpresses immune regulatory proteins such as CD200 and CD276 (B7H3), which is not regulated by the EWS-WT1 fusion protein [81]. Currently, enoblituzumab is being tested in many solid tumors (ClinicalTrials.gov Identifier: NCT02982941), including pediatric patients with DRSCR, the results of this trial is pending. It has previously described that DSRCT shows high expression of CD276 (B7H3) [81] and it was the basis for a phase I trial designed to evaluate the safety, pharmacokinetics and bio-distribution of intra-peritoneal radio-immunotherapy with a monoclonal antibody anti-B7H3 ^131^I-omburtamab in patients with DSRCT or other B7H3 positive neoplasms with peritoneal involvement. The results with 48 treated patients with DSRCT showed this approach is tolerable and the maximum tolerated dose was not reached [130]. A phase II trial was advocated based on these results.

The neurotrophic tyrosine kinase receptor (NTKR) fusions act as driver mutation is a myriad of neoplasms. The use of NTRK inhibitors has demonstrated robust activity across many histologies [131]. Sarcoma was one of the most common tumor type included in the larotrectinib trials [132] and the search for NTRK fusion in sarcoma patients are recommended based on priority criteria [131]. Recently, it was demonstrated that *EWS-WT1* promotes direct NTRK3 transcription and in vivo and in vitro inhibition of NTRK3 cells with entrectinib resulted in significant decrease in cell growth. This finding opens the possibility for future trials with NTRK inhibitor in DSRCT [133].

## 5. Conclusions

In summary, DSRCT is a rare and aggressive disease and fatal for the majority of the patients. A modest improvement in survival has been observed in more recent studies. For second-line treatment, there is no standard of treatment and the results with conventional chemotherapy and novel agents are disappointing. A better understanding of disease biology has identified potential targets to be explored in future clinical trials. It is paramount the work of the cooperative group to organize prospective databanks and conduct clinical trials.

## Figures and Tables

**Table 1:** Impact of Radiation Therapy after surgical resection in the treatment of desmoplastic small round cell tumor (DSRCT).

Author	Year	*N*	% Surgery + Radiation (*N*)	RdT Dose (Gy)	Comparison Group (RdT × no-RdT)	Outcome with Addition of Radiation
Lal et al. [22]	2005	66	43% (29)	30	yes	Improved 3 year OS (55 × 27%, *p* < 0.02)
Forlenza et al. [56]	2015	19	89% (17)	30	no	5-year OS 16%
Stiles et al. [23]	2018	102	20% (21)	NR	yes	No difference in median OS (27.5 × 28.8 mo, *p* = 0.32)
Subbiah et al. [46]	2018	165	42% (69)	NR	yes	No improvement in OS (3.6 × 2.9, *p* = 0.38)
Honore et al. [5]	2019	100	26% (26)	30	yes	Improved survival and cure rate (HR = 0.36, *p* = 0.00013)
Atallah et al. [54]	2016	49 *	55% (27)	20–33	yes	Improved median PPFS (22.5 × 14.2 mo, *p* = 0.024), no significant difference in OS (*p* = 0.40)
Campos et al. [57]	2020	19	21% (4)	30	no	5-year OS 12%

RdT—radiation therapy; NR—not reported; mo—months; OS—overall survival; PPFS—peritoneal progression-free survival; * only patients achieving complete cytoreductive surgery.

**Table 2:** Summary of studies with multimodal therapy for first-line treatment of patients with desmoplastic small round cell tumor.

Author	Year	N	Study Design/Region	Therapy	Overall Survival	Relapse
Lal et al. [22]	2005	66	Retrospective, single center, USA	ChT, CRS, RdT	5-year 15%	NR
Forlenza et al. [56]	2015	19	Prospective, single center, USA	ChT, CRS, BMT, RdT	5-year 16%	3-year EFS 11.0%
Osborne et al. [60]	2015	32	Retrospective, Single center USA	ChT, CRS, RdT	5-year 38%	3-year EFS 9.9%
Honore et al. [61]	2017	48	Retrospective	ChT, CRS, HIPEC, RdT	5-year 19%	5-year DFS 12%
Scheer et al. [52]	2018	60	Prospective, multicenter, Germany, Poland, Austria, Sweden, Switzerland	ChT, CRS, BMT, RdT HIPEC	3-year 30%	3-year EFS 11.0%
Stiles et al. [23]	2018	125	Retrospective, multicenter, USA	ChT, CRS, BMT HIPEC RdT	5-year 10%	NR
Subbiah et al. [46]	2018	165	Retrospective, single center, USA	ChT, CRS, BMT, HIPEC, RdT	5-year 25%	NR
Honore et al. [5]	2019	100	Retrospective, multicenter, France	ChT, CRS, HIPEC, RdT	5-year 5%	3-year DFS 7.0%
Campos et al. [57]	2020	19	Retrospective, single center, Brazil	ChT, CRS, HIPEC RdT	5-year 12%	Median DFS 10 months

ChT—chemotherapy, CRS—cytoreductive surgery, BMT—autologous bone marrow transplant, HIPEC—hyperthermic intraperitoneal chemotherapy, RdT—radiation therapy, DFS—disease-free survival, EFS—event-free survival, NR—not reported.

**Table 3:** Selected studies evaluating the efficacy of systemic treatments in patients with DSRCT according to the line of treatment.

Author, Year	Line of Treatment	*N*	Median Age/Range (Years)	Systemic Therapy	Response	Survival
Farhat et al., 1996 [49]	1st line	5	22/16–26	PA(E)VEP *	80% SD 20% CR	mRFS: 6 mo
Bertuzzi et al., 2003 [63]	1st line	10	29/NA	IVE *	50% PR 20% SD 30% PD	NA
Forlenza et al., 2015 [56]	1st line	19	18.5/10–42	P6-protocol *	78% SD 11% MR 11% PD	mPFS = 12.8 mo 3-year OS = 26 ± 10%
Magnan et al., 2017 [64]	1st line	15	NA	ITB	RR = 27%	mTTP = 18.1 mo 3-year OS = 61%
Scheer et al., 2019 [52]	1st line	60	14.5/6–38	P6-protocol * VAIA * CEVAIE *	NA **	P6-protocol: EFS = 12.9 mo VAIA: EFS = 29.4 mo CEVAIE: EFS = 12 mo
Kushner et al., 1996 [50]	1st and 2nd line	12	14/7–22	P6-protocol *	83% PR	mPFS > 12 mo
Bond et al., 2008 [65]	1st line and beyond	10	16/3–29	Imatinib Mesylate	100% PD	NA
Tap et al., 2012 [66]	1st and beyond	16	33/19–63	Ganitumab	6% PR 63% SD	mPFS = 19 mo
Casanova et al., 2004 [67]	2nd line	1	17	Vinorelbine/ Cyclophosphamide	PR	mPFS > 6 mo
Bisogno et al., 2006 [68]	2nd line and beyond	3	10.6/1–18.5	Irinotecan	100% PD	NA
Italiano et al., 2013 [69]	2nd line and beyond	8	23/14–58	Sunitinib	25% PR 37.5% SD 37.5% PD	mPFS > 4 mo
Verret, B. et al., 2017 [70]	2nd line and beyond	6	25/19–52	Trabectedin	33% SD 67% PD	mPFS = 3.2 mo mOS = 4 mo
Menegaz et al., 2018 [71]	2nd line and beyond	38	25/5–48	Pazopanib	3% CR 3% PR 55% SD 38% PD	mPFS = 5.6 mo mOS = 15.7 mo
Tarek et al., 2018 [72]	3rd line and beyond	5	15/11–28	VCT	80% PR 20% SD	mTTP = 8.5 mo
Thijs et al., 2010 [73]	4th line	1	21	Temsirolimus	SD	PFS = 10 mo OS = 13 mo

Chemotherapeutic regimens: PA(E)VEP (cyclophosphamide, etoposide, doxorubicin or epirubicin, and cisplatin); IVE (ifosfamide, vincristine, and epirubicin); P6-protocol (high-dose cyclophosphamide, doxorubicin, and vincristine, alternating with ifosfamide, and etoposide); ITB (irinotecan, temozolomide, and bevacizumab); VAIA (ifosfamide, vincristine, adriamycin, actinomycin D); CEVAIE (ifosfamide, vincristine, actinomycin D, carboplatin, epirubicin and etoposide); VCT (vinorelbine, cyclophosphamide, and temsirolimus). NA: not available; CR: complete response; PR: partial response; MR: mixed response; SD: stable disease; PFS: progression-free survival; OS: overall survival; RFS: relapse-free survival; TTP: time-to-progression; EFS: event--free survival; RR: response rate; mo: months; m: median. * After induction chemotherapy, patients underwent additional therapies, which could include myeloablative chemotherapy followed by autologous stem cell transplant, surgery, and/or radiotherapy. ** VAIA scheme correlated with an increased chance of R0 or R0/R1 resection.

**Table 4:** Selected trials including desmoplastic small round cell tumor (DSRCT) patients.

Phase of Trial	Design	Primary Outcome	ClinicalTrials.gov Identifier
Phase 1/2	Ramucirumab IV + Cyclophosphamide p.o. + Vinorelbine IV (experimental arm), versus Cyclophosphamide p.o. + Vinorelbine IV	1. Progression-fFree survival	NCT04145349
Phase 1	2 cycles of the investigational combination irinotecan, temozolomide and bevacizumab, will be given followed by conventional chemotherapy with a modified P6 approach and surgical local control. Completion of modified P6 chemotherapy will be followed by a second-look surgery.	1. Tolerability 2. Adverse event profile	NCT01189643
Phase 2	Experimental arm A: Single dose of IP RIT administered through an IP catheter with 131 I-omburtamab at 80 mCi/m^2^, followed by WA-IMRT approximately 2–4 weeks after completing IP RIT Experimental arm B: Single dose of IP RIT administered through an IP catheter with 131 I-omburtamab at 80 mCi/m^2^ Experimental arm C: Single dose of IP RIT administered through an IP catheter with 131 I-omburtamab at 80 mCi/m^2^	1. Progression-free survival	NCT04022213
Phase 1/2	Dose Escalation/Dose Expansion Study of Prexasertib in Combination with Irinotecan 15 mg/m^2^ IV daily × 10 days in 21 day cycles	1. Recommended phase II does of Prexasertib 2. Response	NCT04095221
Phase 2	Nab-paclitaxel will be administered as follows: Age ≥ 21: 125 mg/m^2^ days 1, 8 and 15 in cycles of 28 days Age ≥ 6 months and ≤ 20 years: 240 mg/m^2^ (for patients weighing > 10 kg) and 11.5 mg/kg (for patients weighing ≤ 10 kg) on days 1, 8 and 15 in cycles of 28 days	1. Overall response rate 2. Objective response rate	NCT03275818
Phase 2	Participants will receive vincristine, doxorubicin, cyclophosphamide, ifosfamide, etoposide, irinotecan, temozolomide, temsirolimus, bevacizumab, and sorafenib. Depending on the size and location of the participant’s tumor, they will have surgery alone, radiation alone or surgery followed by radiation.	Participants with DSRCT will not be included in the analysis of primary outcome	NCT01946529
Phase 2	Allogeneic Hematopoietic Stem Cell Transplantation	1. Transplant-related mortality 2. Rate of grade III or higher organ toxicity attributable to conditioning	NCT04530487
Phase 1	Patients undergo cytoreduction and HIPEC over 60 min consisting of doxorubicin and cisplatin. Patients then receive sodium thiosulfate IV over 12 h.	1. To assess the feasibility of HIPEC with doxorubicin and cisplatin after surgical resection. 2. To assess morbidity, hospital length of stay and peri-operative mortality outcome.	NCT04213794
Phase 1	Experimental arm A: participants will receive B7H3-specific CAR T cells only Experimental arm B: participants will receive CAR T cells directed at B7H3 and CD19	1. Safety and tolerability 2. Determine the MTD 3. Assess the DLT and describe the full toxicity profile 4. Assess the feasibility of manufacturing B7H3 and B7H3xCD19 specific CARs	NCT04483778
Phase 1	Experimental arm A: participants will receive EGFR-specific CAR T cells only. Experimental arm B: participants will receive CAR T cells directed at EGFR and CD19	1. Estimate the MTD and DLT 2. Assess the number of successfully manufactured EGFR806 and EGFR806xCD19 CAR T cell products 3. Safety	NCT03618381
Phase 2	Nivolumab 240 mg IV every 2 weeks plus Ipilimumab 1 mg/m^2^ IV every 6 weeks	1. Response to therapy as evaluated by RECIST 1.1	NCT02982486
Phase 2	Reduced-intensity chemotherapy, haploidentical bone marrow, post-transplant cyclophosphamide and shortened duration tacrolimus	1. Safety	NCT01804634
Phase 1	CLR 131 intravenous administration	1. Number of participants with DLT	NCT03478462

IV: intravenous; IP RIT: intraperitoneal radioimmunotherapy; MDT: maximum tolerated dose; DLT: dose limiting toxicity.

## Data Availability

No new data were created or analyzed in this study. Data sharing is not applicable to this article.
